# Electrokinetically driven continuous-flow enrichment of colloidal particles by Joule heating induced temperature gradient focusing in a convergent-divergent microfluidic structure

**DOI:** 10.1038/s41598-017-11473-w

**Published:** 2017-09-07

**Authors:** Cunlu Zhao, Zhengwei Ge, Yongxin Song, Chun Yang

**Affiliations:** 1 Key Laboratory of Thermo-Fluid Science and Engineering of MOE, School of Energy and Power Engineering, Xi’an Jiaotong University, Xi’an, 710049 China; 2 School of Mechanical and Aerospace Engineering, Nanyang Technological University, 50 Nanyang Avenue, Singapore, 639798 Singapore; 3 Department of Marine Engineering, Dalian Maritime University, 1 Linghai Road, Dalian, 116026 China

## Abstract

Enrichment of colloidal particles in continuous flow has not only numerous applications but also poses a great challenge in controlling physical forces that are required for achieving particle enrichment. Here, we for the first time experimentally demonstrate the electrokinetically-driven continuous-flow enrichment of colloidal particles with Joule heating induced temperature gradient focusing (TGF) in a microfluidic convergent-divergent structure. We consider four mechanisms of particle transport, i.e., advection due to electroosmosis, electrophoresis, dielectrophoresis and, and further clarify their roles in the particle enrichment. It is experimentally determined and numerically verified that the particle thermophoresis plays dominant roles in enrichment of all particle sizes considered in this study and the combined effect of electroosmosis-induced advection and electrophoresis is mainly to transport particles to the zone of enrichment. Specifically, the enrichment of particles is achieved with combined DC and AC voltages rather than a sole DC or AC voltage. A numerical model is formulated with consideration of the abovementioned four mechanisms, and the model can rationalize the experimental observations. Particularly, our analysis of numerical and experimental results indicates that thermophoresis which is usually an overlooked mechanism of material transport is crucial for the successful electrokinetic enrichment of particles with Joule heating induced TGF.

## Introduction

Microfluidic technologies have been widely demonstrated with the potential of accurate and fast analyses/diagnoses in biological and chemical sciences as well as drug discovery^1–3^. Basically, microfluidics aims to integrate multiple steps of analyte manipulations in conventional analyses, usually consisting of sample concentration, pumping, mixing, chemical reactions, separation, detection and so on, into a single microchip. Therefore, microfluidics-based analytical devices gain various kinds of advantages over the conventional large-scale analytical systems, such as low analyte consumption and costs, fast analyses, good portability as well as high throughput etc. However, due to extremely small volumes of analytes (nanoliter or less) handled in microfluidic analyses, original low concentration of analytes would lead to poor detection which is a critical challenge faced by the microfluidics-based analytical systems^4–7^. To overcome this challenge has necessitated the pre-concentration of dilute samples prior to further manipulations, detection and analysis.

Generally, the methods for sample concentration can be divided into two large categories, non-electrokinetic methods and electrokinetic methods^8^. The popular non-electrokinetic methods include immunocapture based trapping^9^, magnetic beads assisted trapping^10^, thermophoretic trapping^11^ etc. Recently, there is also an emerging non-electrokinetic method which utilizes hydrodynamic inertia to enrich particulate analyte^12–14^. The electrokinetic methods also have been developed for analyte concentration in microfluidics. The advantages of electrokinetic methods include ease of control and applicability to a wide range of samples. One group of the electrokinetic concentrating techniques is the stacking method. In this technique, analytes migrate from one zone of higher velocity into another zone of lower velocity in which analytes become concentrated. The velocity change can be produced by two buffer solutions with different conductivities, such as field amplified sample stacking method^15, 16^ and isotachophoresis^17–19^. Although the concentration enhancement by the stacking method can be fairly high^20, 21^, it has several drawbacks, such as the need of multiple buffers, the requirement of relatively long channels, the limited concentration enhancement due to the insufficient difference between two buffer velocities, and the difficulty in controlling the concentrated samples because of their dynamic motion etc. Another group of electrokinetic analyte concentration technique is termed the field gradient focusing method, in which analytes are enriched at a unique equilibrium point where the net velocity of analytes is zero. The point of zero net velocity can be produced by pH gradient in the isoelectric focusing^22, 23^ or electric field gradient in the electric field gradient focusing^24–26^. The isoelectric focusing can achieve a relatively high concentration enhancement of 1000-fold as shown by both mass spectrometry analysis and direct imaging^27^. The isoelectric focusing however is limited to analytes with accessible isoelectric points. Electric field gradient focusing which manipulates the electric field in channels by patterning the shape and location of electrodes, usually involves complex design and tedious fabrication for electrodes. In addition to aforementioned two groups of electrokinetic methods, other electrokinetic concentration techniques in microfluidics include the trapping methods which make use of the concentration polarization near ion-selective membranes/nanofilters^28–32^, electrokinetic microvortices^33, 34^ and dielectrophoresis^35, 36^. Although the technique of ion-selective membranes can produce a concentration enhancement as high as million-fold^29^, it requires the sophisticated and expensive nanotechnology to fabricate nanochannels. The microvortices method involves the tedious surface treatment for modulation of surface charge or zeta potentials. The dielectrophoresis method produces limited concentration enrichment as well as requires sophisticated fabrication of complex electrode patterns.

Temperature gradient focusing (TGF) is a relatively new electrokinetic method for sample concentration in microfluidics. Essentially, TGF is one category of the field gradient focusing method. The point of zero velocity (focusing point) in TGF is produced by the velocity gradient due to the presence of a temperature gradient along the channel^37^. As compared to aforementioned concentration techniques, TGF can achieve the concentration of charged analytes in simple microfluidic structures with relatively short channel length^38^. The concept of TGF has been experimentally achieved for a variety of analytes, including amino acids, DNA and proteins and fluorescent dyes with temperature gradient produced by external heating/cooling equipment via electrical or optical means^38–40^. Usually, the application of electric field inevitably produces Joule heat in the conducting buffer solutions^41^. Then such Joule-heating method in combination with varying cross-section of channel provides an alternative way of setting up the temperature gradient required in TGF^42–44^. In comparison with the external heating mode, the Joule-heating mode does not require bulky external heating units, and thus consumes less power, simplifies the device design and fabrication, and enhances the portability and compactness of device. Recently, with a combined AC and DC electric field, an unprecedentedly high concentration enhancement of charged solute was accomplished with the Joule-heating mode of TGF^45^.

Noteworthily, Joule-heating mode of TGF so far has been only utilized for concentration of solutes. Practical applications frequently demand the concentration of particulate materials, such as the enrichment of bioparticles^12, 46^ for enhancing analysis sensitivity and the enrichment of colloidal particles^34, 47^ for colloid crystal assembly. Moreover, the existence of temperature gradient inevitably leads to thermophoresis which introduces another new mechanism of material transport neglected in the existing studies of TGF. In this paper, we report the electrokinetic enrichment of colloidal particles using Joule-heating induced TGF for the first time. We further demonstrate that dielectrophoresis and thermophoresis both play important roles in the enrichment of particles. Parametric studies under various experimental conditions (effects of electric field and particle size) are performed for examining the TGF performance. In addition, we formulate a multiphysical model to numerically simulate the experimental observations, and find that the modeling results can well account for the mechanisms of particle enrichment.

## Results and Discussion

### Effect of applied voltage on the particle enrichment

**Figure 1:**
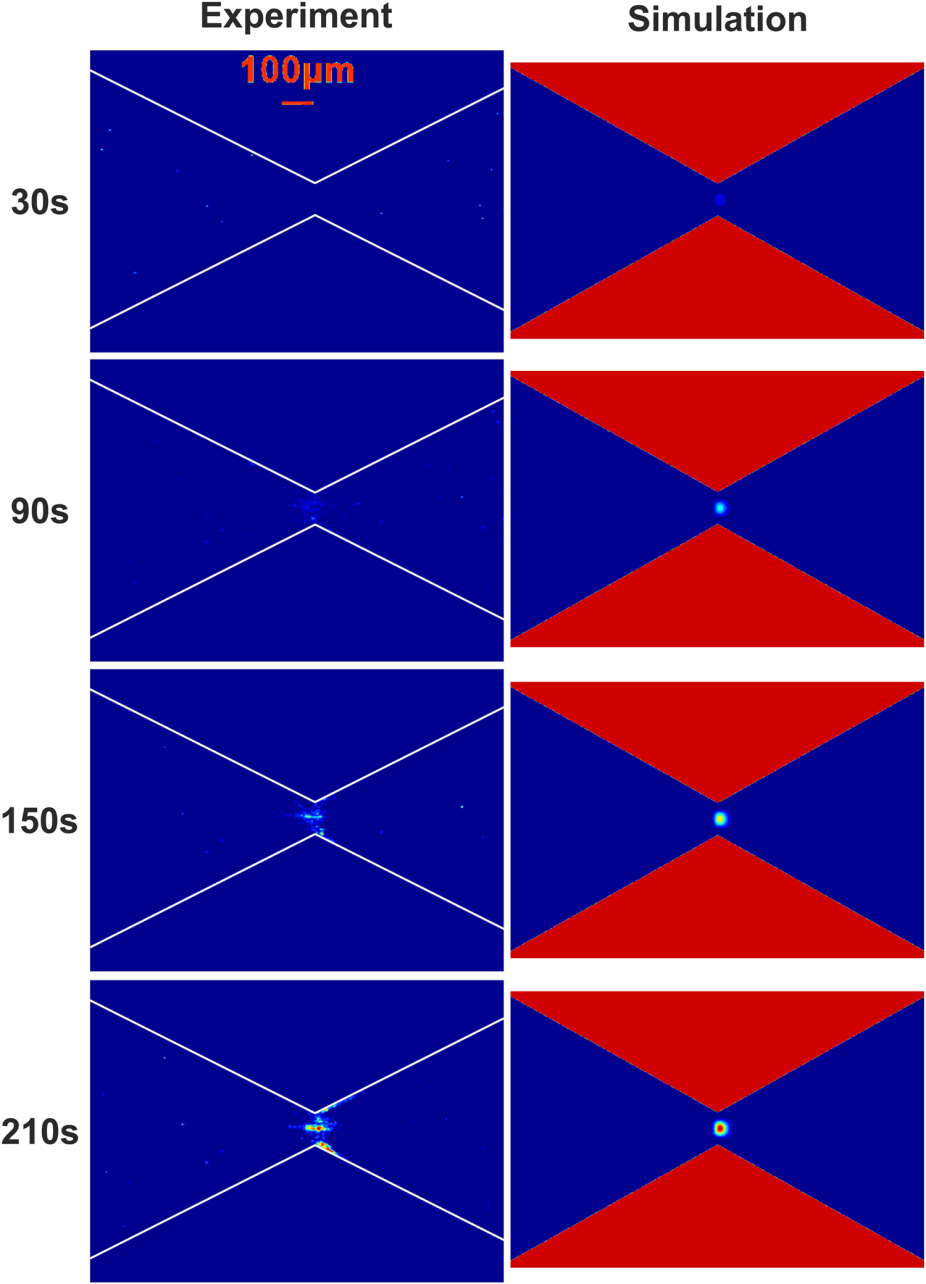
Transient enrichment of 1 µm fluorescent particles dispersed in the 180 mM Tris-borate buffer solution at the constriction region of the convergent-divergent microfluidic structure under a combined voltage of 700 V DC + 400 sin(2π*f*t)V AC (*f* = 10 kHz). The images in the left column show the contours of fluorescent intensity of 1 μm particles observed at four different time instants, and the images in the right column show the numerically simulated concentration contours of 1 μm particles at the corresponding time instants. In the numerical simulation, the following parameters are used: the zeta potentials of channel walls and particles at room temperature *ζ*_w_(T_0_) = −25 mV and *ζ*_p_(T_0_) = −26 mV respectively, and the Soret coefficient of particles S_T_ = −1.25.

The images shown in Fig. 1 are the time evolution of enrichment of 1 µm particles dispersed in a 180 mM Tris-borate buffer under the effect of combined voltages of 700 V DC and 400 sin(2π*f*t) V AC with *f* = 10 kHz. The contour images of measured fluorescent intensity in the left column indicate that the particles are enriched gradually near the narrowest constriction of the microfluidic channel. The images in the right column are the numerically simulated concentration of particles at four time instants corresponding to the experimental observations in the left column. Since the temperature gradient at the narrowest constriction of the channel is high so that the effect of thermophoresis of particles is expected, and a Soret coefficient of S_T_ = −1.25 experimentally obtained for 1 µm particles was used in the numerical simulation^48^. Overall, the numerical model can reasonably describe the observed transient particle enrichment achieved by the Joule heating induced TGF. However, it should be noted that the numerical model fails to predict the deposition of particles on the walls near the constriction; this is because the numerical model presented in this study is for describing particle enrichment only, but not for describing particle deposition.

**Figure 2:**
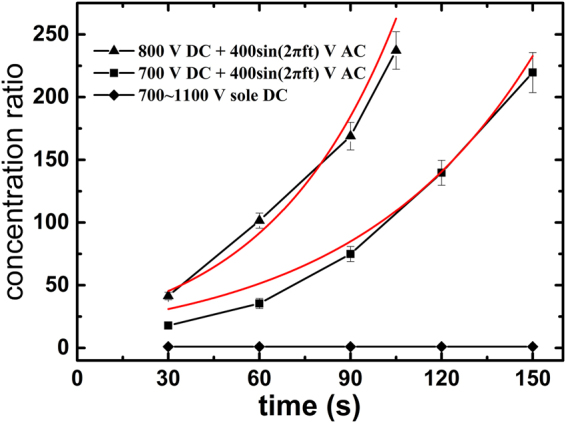
Variation of the concentration ratio ($\bar{C}/{C}_{0}$) of 1 µm particles with time under three modes of applied electric voltages: (i) sole DC voltages varying from 700 to 1100 V; (ii) combined 700 V DC + 400 sin(2π*f*t)V AC; (iii) combined 800 V DC + 400 sin(2π*f*t)V AC (*f* = 10 kHz in both (ii) and (iii)). It shows no particle enrichment under a sole DC within the indicated range. About 200-fold concentration enhancement of particles was achieved within 100 s under the case (iii). The lines with symbols are experimental results, and the smooth solid lines (red) are numerical results.

The particle enhancement can be characterized quantitatively by the particle concentration ratio which is defined as $\bar{C}/{C}_{0}$ with $\bar{C}$ being the averaged particle concentration across the narrowest constriction and C_0_ being the original particle concentration of testing samples. Figure 2 presents the concentration ratios of 1 µm particles at the narrowest constriction under three different cases of applied voltages. Our experiments find that for a fixed 400 V AC amplitude, there is a DC voltage window (from 600 V to 900 V) in which the enrichment of particles can be achieved and also becomes enhanced as the DC voltage increases (see the concentration enhancement from 700 V DC to 800 V DC in Fig. 2). No enrichment of particles occurs as the DC voltage falls outside of this window (either smaller than 600 V or larger than 900 V). For a fixed AC voltage, the DC voltage governs the following two effects: (i) The temperature and temperature gradient because of Joule heating, (ii) The particle transport by the combined action of electrophoresis and electroosmosis. Decrease of the DC voltage reduces the enrichment of particles because on the one hand the induced temperature gradient becomes smaller and thus suppresses the TGF, and on the other hand, less particles are transported to the constriction for enrichment. As the DC voltage drops below 600 V, effects of both (i) and (ii) are significantly reduced such that the particle enrichment vanishes. However, increase of the abovementioned two effects due to increasing DC voltage does not always contribute positively to the enrichment of particles. It should be noted that there is a competition between the abovementioned two effects as the DC voltage increases. Inside the DC window, an increase of effect (i) with the DC voltage dominates over that of effect (ii), leading to the enhancement of TGF and the associated particle enrichment; while further increasing the DC voltage above 900 V, the increment of effect (ii) predominates, and then particles move so fast through the constriction that the TGF is not strong enough to confine the particles to the constriction region.

Figure 2 also shows a nearly 200-fold concentration enhancement of particles within 100 s under a combined voltage of 800 V DC and 400 sin(2π*f*t)V AC with *f* = 10 kHz. However, no particle enrichment was observed under sole applied DC electric voltage, even when the DC voltage was increased to 1100 V which generates much higher temperature and temperature gradient than the case of a combined voltage of 700 V DC and 400 sin(2π*f*t)V AC with *f* = 10 kHz. Moreover, it was observed that under our experimental conditions all particles migrate from the left to the right, indicating that the electrophoretic motion of particles dominates over the TGF enrichment of particles. In order to achieve enrichment of particles, the DC electric field (associated with the electrophoretic velocity of particles) should be reduced while maintaining a sufficient temperature gradient for the TGF. Hence, with a combined AC and DC electric field, the AC component contributes to producing the temperature gradient via Joule-heating effect, and thus a lower DC voltage is preferred.

**Figure 3:**
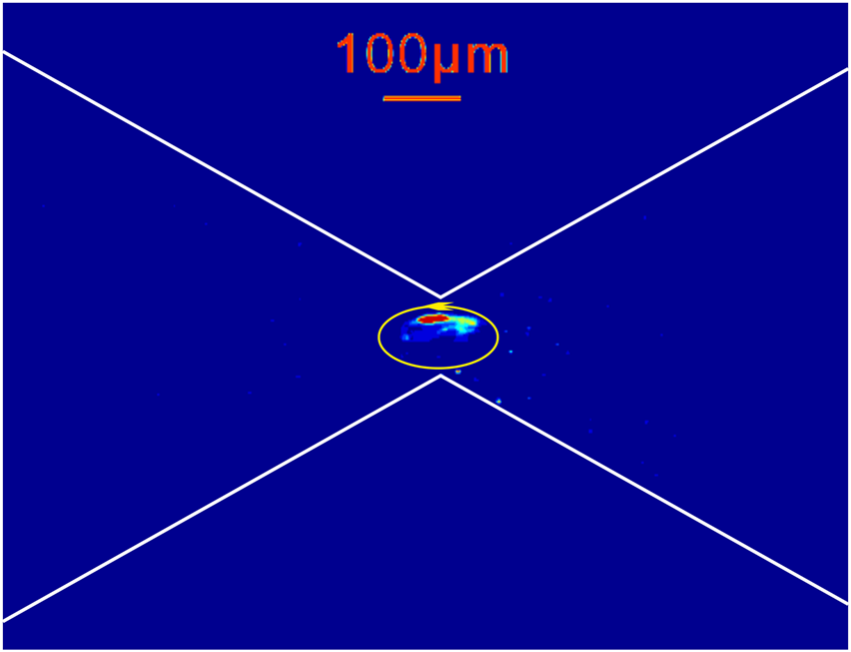
A contour image of the fluorescent intensity for the solution of 1 µm particles near the convergent-divergent microfluidic structure after imposing a combined voltage of 700 V DC + 400 sin(2π*f*t)V AC with a frequency *f* = 300 Hz for 120 s. With such a low frequency, a counterclockwise vortex induced by the bulk electroosmotic flow was observed in the experiment.

In addition, shown in Fig. 3 is the presence of a counterclockwise vortex near the constriction, and such vortex tends to dispel the enriched particles away from the constriction as the applied AC frequency is lowered to the order of 10^2^ Hz. As discussed earlier, if the AC frequency is high (such as 10 kHz), the bulk flow is only due to the DC voltage driven electroosmosis. However, as the AC frequency decreases to the order of 10^2^ Hz, the time period of AC electric field under such circumstance is comparable to the characteristic response time of the bulk electroosmosis flow in the microchannel (${\tau }_{eof}=O({10}^{-2}){\rm{s}}$)^49^. Hence, such low frequency AC field allows the development of AC field driven electroosmosis, leading to the formation of vortical flows near the constriction as shown in Fig. 3 (see the corresponding movie in Supplementary Information). Furthermore, the electrothermal effect^50, 51^ could be another contributing factor to the formation of vortical flow in microfluidic constrictions. The experimental tests also indicate that the intensity of particle enrichment hardly changes when the AC frequency varies from 1 kHz to 100 kHz.

### The role of thermophoresis in the enrichment of submicron particles

**Figure 4:**
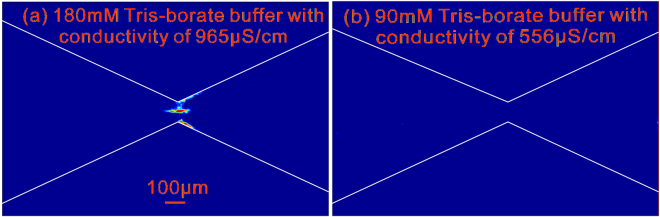
Contour images of the fluorescent intensity of 1 µm particles dispersed in (**a**) 180 mM (conductivity 965 µS/cm with particle enrichment) and (**b**) 90 mM (556 μS/cm without particle enrichment) Tris-borate buffer solutions, after applying a combined voltage of 700 V DC + 400 sin(2π*f*t)VAC with a frequency *f* = 10 kHz for 300 s. It should be noted that according to the theory of dielectrophoresis (i.e., Eq. (6) in Supplementary Information), the dielectrophoretic force exerting on 1 µm particles in both case (**a**) and case (**b**) should be the same, suggesting that the enrichment in case (**a**) is not mainly due to dielectrophoresis.

The strong variation of electric field in microfluidic convergent-divergent structure induces both dielectrophoresis and thermophoresis. Then a question naturally arises - which mechanism dominates the enrichment of small particles (such as 1 µm particles)? We carried out a controlled experiment to answer this question, and the experimental results are shown in Fig. 4. Since the conductivity of buffer solution is much larger than that of particles in present experiments, the dielectrophoretic force is independent of buffer concentration as suggested by Eq. (6) in Supplementary Information. Presumably, if the enrichment of 1 µm particles dispersed in the 180 mM Tris-borate buffer solution (Fig. 4(a) and its corresponding movie in Supplementary Information) is caused by dielectrophoretic trapping, the enrichment phenomena naturally should also be observable for particles dispersed in the Tris-borate buffer solution with a lower concentration. However, Fig. 4(b) amply shows no enrichment of particles in the 90 mM Tris-borate solution (see the corresponding movie in Supplementary Information). Hence, it is affirmative that the enrichment of smaller particles (1 µm particles) is not due to the dielectrophoresis. On the other hand, it should be noted that the reduction of buffer concentration (equivalent to the reduction of buffer conductivity) results in less Joule heat which could not sustain TGF of particles. So the absence of enrichment of particles in Fig. 4(b) verifies that the Joule-heating mode of TGF, instead of the dielectrophoresis, plays a dominant role in the concentration of 1 µm particles.

**Figure 5:**
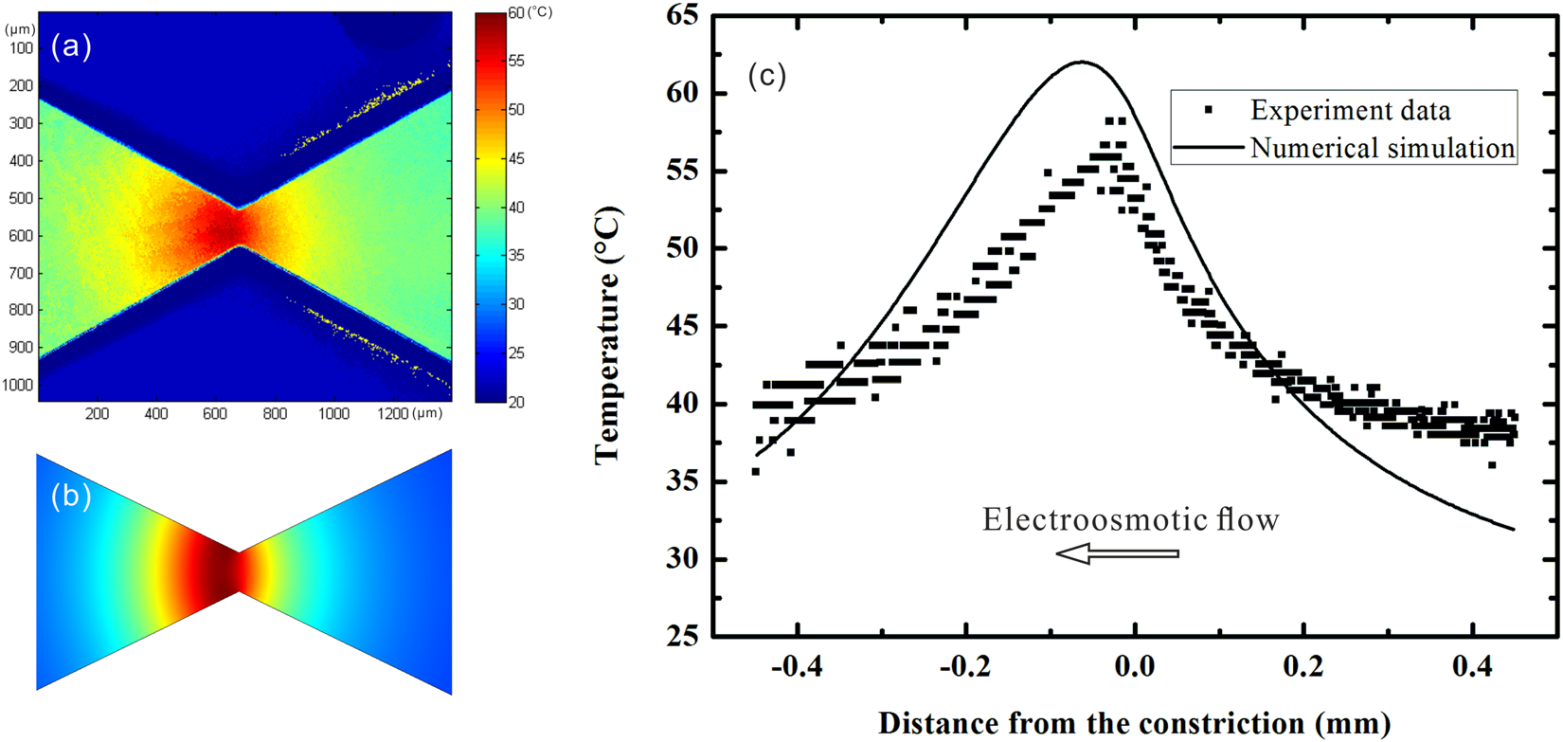
Temperature distributions at the constriction of the convergent-divergent microfluidic structure under a combined voltage of 700 V DC + 400 sin(2π*f*t)V AC with *f* = 10 kHz. (**a**) Experimentally measured temperature contour near the constriction section; (**b**) Numerically simulated temperature contour near the constriction; (**c**) Comparison between the numerically simulated and experimentally measured solution temperature profiles along the centerline of the microfluidic structure.

In order to clarify the role of thermophoresis in the enrichment of particles, the information of temperature field near the constriction is required. We thus measured the temperature distribution in the 180 mM Tris-borate buffer solution around the constriction region by using the Rhodamine B based thermometry whose principle is based on a sensitive temperature dependence of fluorescent intensity of Rhodamine B^52^. The general trend is that a decreasing Rhodamine B fluorescent intensity indicates an increasing solution temperature. In this experiment, the concentration of Rhodamine B in the solution was 0.1 mM, and the solution temperature was measured by using a calibrated relationship between temperature and fluorescent intensity reported previously^48^. Figure 5 shows the results of both temperature measurements and numerical predictions (note: the numerical model is presented in Supplementary Information). An inherent Joule heating induced temperature gradient is generated in the microchannel due to varying cross-sectional area of the channel. Expectedly, the temperature near the narrowest region of the channel is highest because of the largest electric current density, as is shown in Fig. 5(a) and (b). It is also noted that the variation of temperature mainly along the axial direction of the microchannel. Though there is temperature difference along the transversal direction of the channel, such difference is quite small because the channel is made from PDMS which has a much lower thermal conductivity than the buffer solution has. However, the temperature distribution around the constriction region is asymmetric which results from electroosmotic-flow-induced convective heat transfer from the right to the left. Figure 5(c) shows that despite certain difference between the numerical predictions and the experimental measurements, the numerical model can well capture the experimental trend. The deviation of the numerical model from the experiment could be due to some assumptions such as the uniform equivalent heat transfer coefficient and parameters such as zeta potential and other properties used in the numerical model as well as experimental errors. It is noted that the measured temperature gradient near the constriction is determined to be 1.5 × 10^5^ K/m. Such a high temperature gradient usually gives to appreciable thermophoresis^53, 54^ which can play an important role in the TGF of particles.

**Figure 6:**
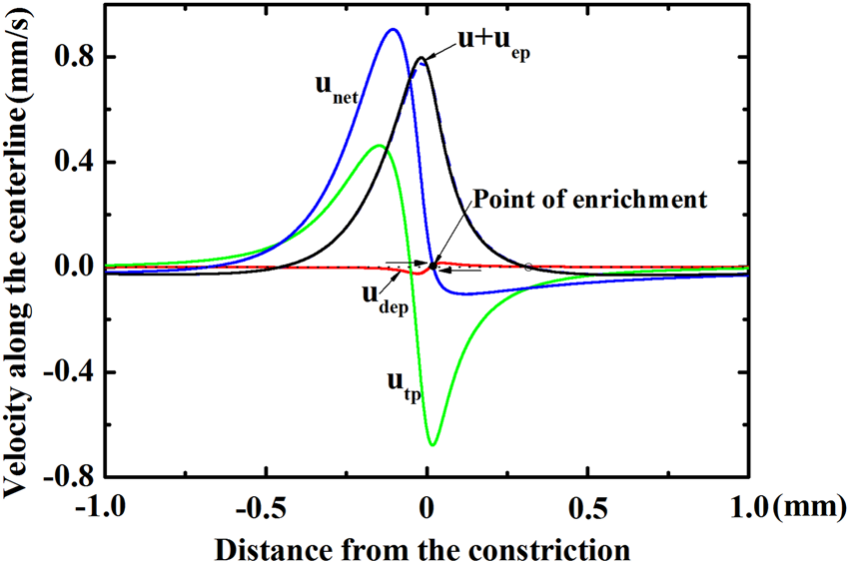
Numerically computed axial velocities of 1 µm particles due to various mechanisms of transport along the centerline of the convergent-divergent microfluidic structure under a combined voltage of 700 V DC + 400 sin(2π*f*t)V AC with *f* = 10 kHz. In the plot, u, u_ep_, u_dep_, u_tp_ are the horizontal components of velocity vectors $\overrightarrow{u}$, ${\overrightarrow{u}}_{ep}$, ${\overrightarrow{u}}_{dep}$ and ${\overrightarrow{u}}_{tp}$ in Eq. (1) of Supplementary Information, respectively, and u_net_ = u + u_ep_ + u_dep_ + u_tp_ is the total net velocity. The horizontal dot line denotes zero velocity, and the dash line is the net velocity of particles without consideration of thermophoretic transport. The solid circular symbol shows the point of enrichment with consideration of all four transport mechanisms, and the empty circular symbol shows the point of enrichment without consideration of thermophoretic transport.

To explore the detailed mechanisms of the particle enrichment in Joule heating induced TGF, we take advantages of the proposed numerical model to compute the profiles of axial velocities of 1 µm particles, induced by various mechanisms of particle transport along the centerline of the convergent-divergent structure, and the results are presented in Fig. 6. In the TGF, the particles are enriched at the location where the net velocity of particles becomes zero. Such zero net velocity of particles *u*_net_ is predicted very near the constriction (see the solid dot in Fig. 6) and it leads to a continuous accumulation of the particles coming from both sides of the convergent-divergent channel. In other words, the zero-velocity point primarily determines the location of particle enrichment, and thus is also termed the *point of enrichment*. Furthermore, Fig. 6 shows that both the bulk electroosmosis and the electrophoresis (u + u_ep_) transport the particles to the narrowest constriction where the thermophoretic velocity u_tp_ becomes significant to trap the particles. Here for 1μm particles the dielectrophoretic velocity u_dep_ plays an insignificant role; this is also in accord with the experimental observations in Fig. 4. In addition, for a comparison the figure also includes the profile of net velocity without inclusion of thermophoresis (see the dash line), and it is shown that the zero-velocity point or the enrichemet zone (see the hollow dot) shifts away from the narrowest constriction, which clearly differs from the experimental observation shown in Fig. 4(a).

**Figure 7:**
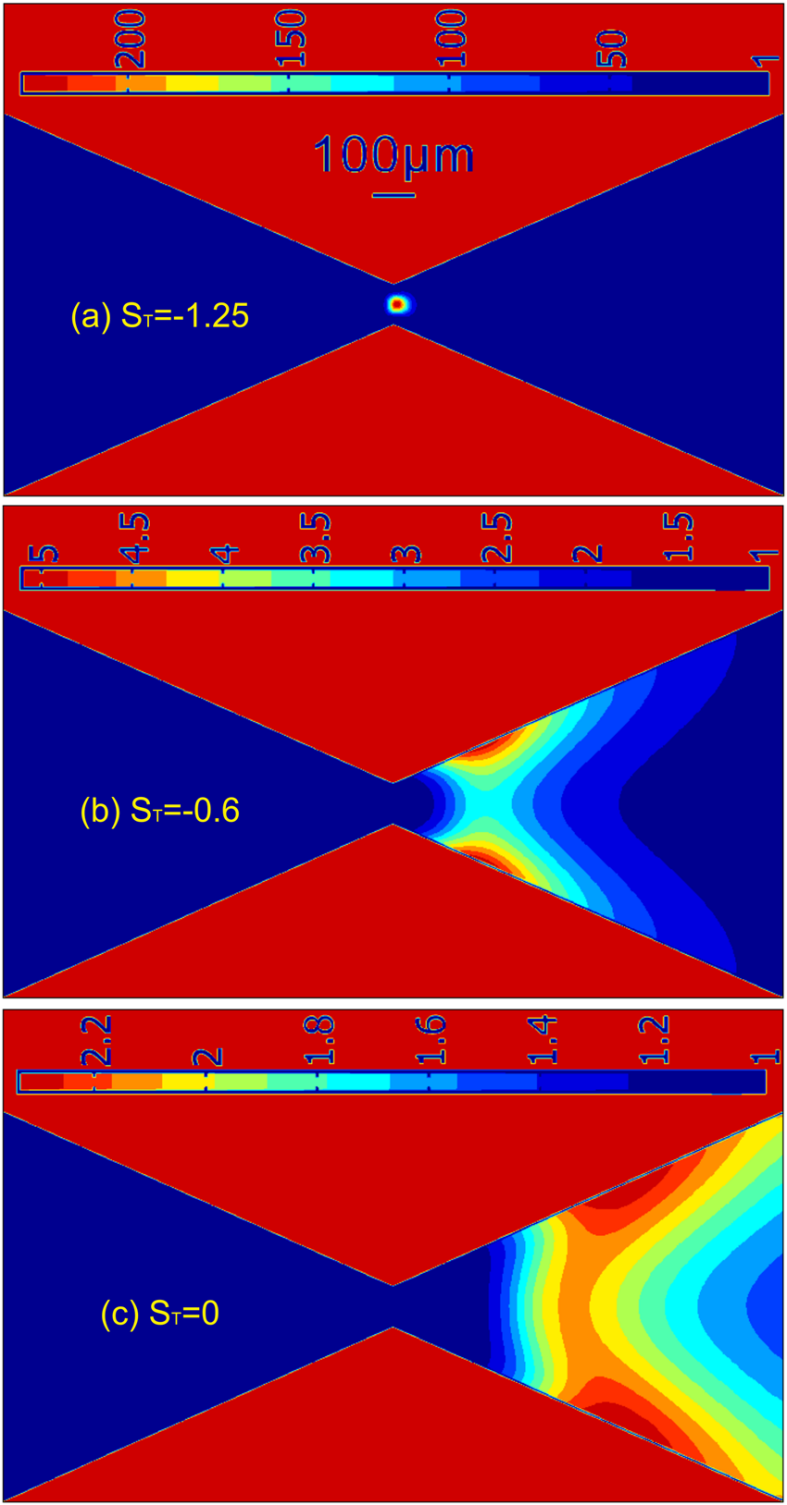
Effect of thermophoresis on the enrichment of 1 µm particles. Numerically computed contours of the concentration enhancement (C/C_0_) of 1 µm particles are for three different strengths of thermophoresis, including (**a**) S_T_ = −1.25, (**b**) S_T_ = −0.6 and (**c**) S_T_ = 0 after imposing a combined voltage of 700 V DC + 400 sin(2π*f*t)V AC with *f* = 10 kHz for 150 s.

Figure 6 already reveals an important role of the thermophoresis in determining the location of particle enrichment due to TGF. However, one still cannot figure out how thermophoresis would affect the particle enchantment quantitatively. As discussed early, the Joule heating induced TGF of 1 µm particles mainly results from several mechanisms (electroosmosis, electrophoresis and thermophoresis), and thus it is experimentally impossible to separate thermophoresis from other mechanisms because all mechanisms are highly coupled via temperature. Fortunately, the numerical simulation provides a way of identifying the effect of thermophoresis on particle enrichment by simply varying the magnitude of Soret coefficient. The simulation results of the concentration enhancement of 1 µm particles for three different strengths of thermophoretic effect are shown in Fig. 7. Our previous work showed that for the particles concentrated near the constriction, negative Soret coefficients (thermophilic behavior) are expected^48^. Figure 7(a) presents the concentration enhancement of particles after 150 s for S_T_ = −1.25. The average concentration ratio of particles across the narrowest constriction is around 230, which is very close to the experimentally measured value of 219 in Fig. 2. Furthermore, the location and shape of the zone of particle enrichment are in reasonably good agreement with the experimental observations at 150 s shown in Fig. 1. As the magnitude of Soret coefficient decreases (say S_T_ = −0.6 in Fig. 7(b)), the zone of particle enrichment shifts to the right side of the constriction, and the highest value of the concentration ratio is only about 5 which is significantly lower than the case when S_T_ = −1.25. In absence of thermophoresis effect (say S_T_ = 0 in Fig. 7(c)), the zone of particle enrichment moves further towards the right side of the constriction, and the concentration enhancement is further reduced. The numerical results in Fig. 7 adequately show that the thermophoresis attracts the particles from the low temperature region (the wide region) towards the higher temperature region (the narrow region), thereby leading to the particle enrichment at the constriction section.

### Effect of particle size on the particle enrichment

**Figure 8:**
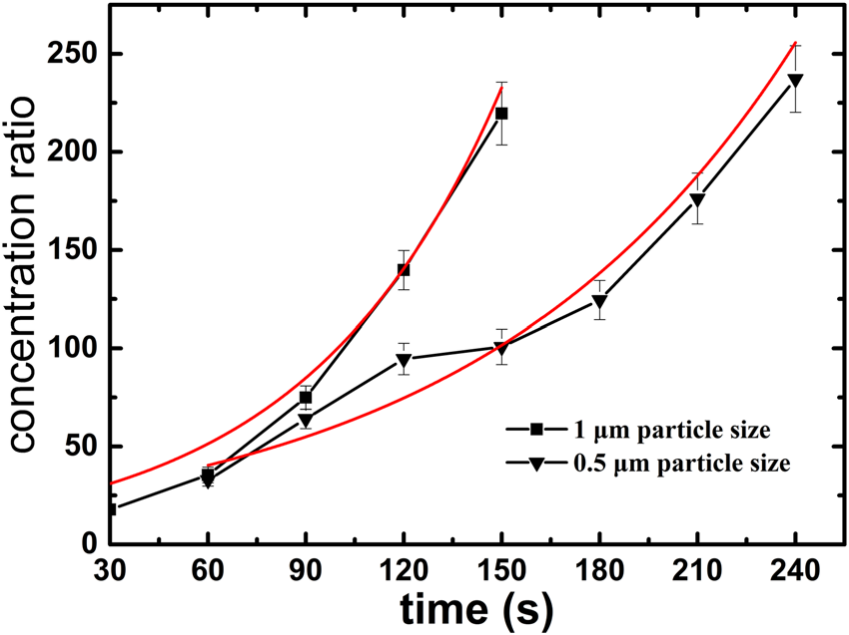
Variation of the concentration ratio $(\bar{C}/{C}_{0})$ of 0.5 µm and 1 µm particles with time under a combined voltage of 700 V DC + 400 sin(2π*f*t)V AC with a frequency *f* = 10 kHz. The lines with symbols are experimental results, and the smooth solid lines (red) are numerical results.

Colloidal particles with three different diameters of 0.5 µm, 1 µm, and 5 µm were used in the experiments. The two smaller particles (0.5 µm and 1 µm) were successfully concentrated under a combined field of 700 V DC and 400 sin(2π*f*t) V AC with *f* = 10 kHz. Figure 8 shows the variation of concentration ratios of 0.5 µm and 1 µm particles with time. It is seen that the concentration enhancement becomes stronger as the particle size increases from 0.5 µm to 1 µm. The magnitude of the Soret coefficient characterizing the strength of thermophoresis is smaller for 0.5 µm particles, as compared to that of 1 µm particles^48^. This suggests that 1 µm particles would acquire a higher velocity which facilitates the enrichment of particles.

**Figure 9:**
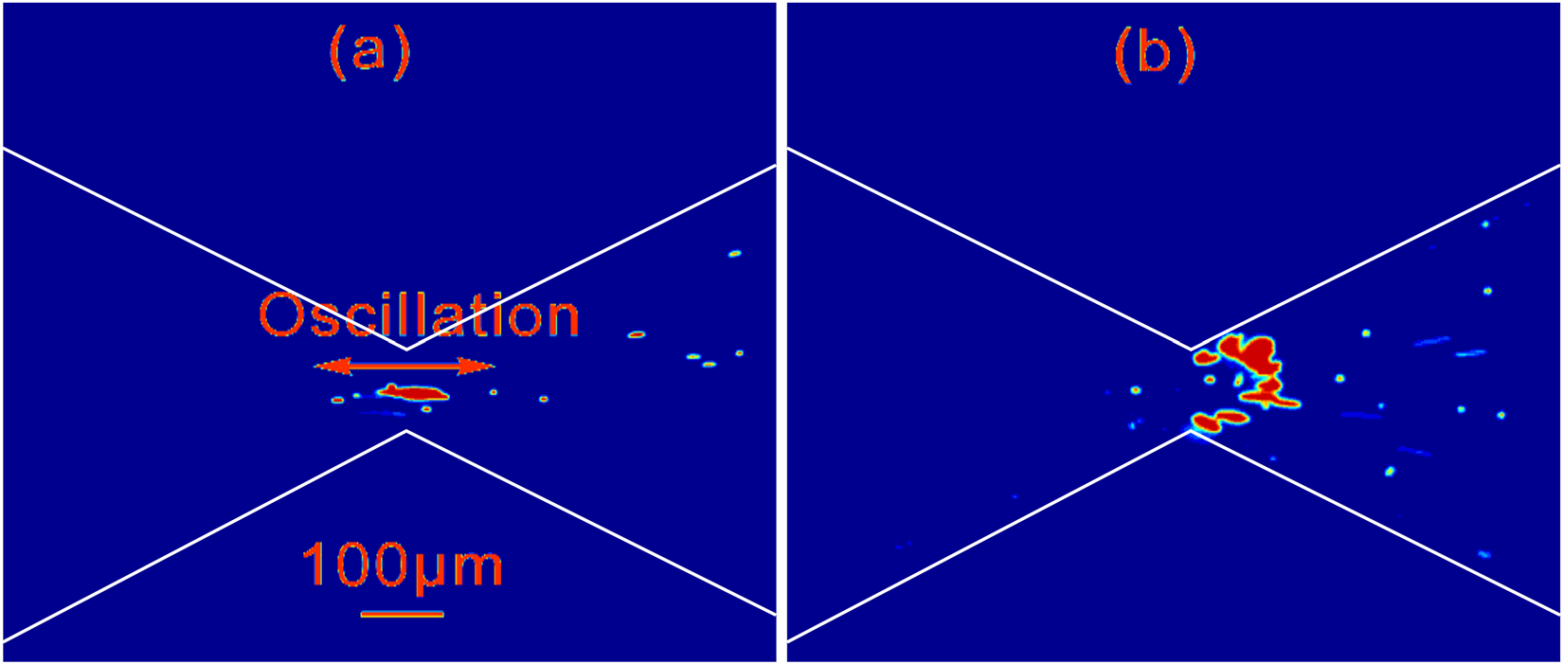
Enrichment behavior of 5 µm fluorescent particles near the convergent-divergent microfluidic structure after 5 minutes of imposing a combined voltage of (**a**) 700 V DC + 400 sin(2π*f*t)V AC (**b**) 800 V DC + 400 sin(2π*f*t)V AC with a frequency *f* of 10 kHz. Case (**a**) shows particles enriched and also oscillating, and Case (**b**) shows particles enriched at the right side of the constriction.

However, with further increasing the particle size from 1 µm to 5 µm and under the same applied voltage, the trapped particles were observed to oscillate within the constriction region, as shown in Fig. 9(a). Here, we perform a qualitative analysis based on the preceding discussion of 1 µm particles to clarify the roles of dielectrophoresis and thermophoresis in the enrichment of 5 µm particles. The conclusions from several previous works^48, 55, 56^ suggested that the Soret coefficient of 5 µm particle is larger than that of 1 µm particle. However, the diffusivity of 5 µm particle is smaller than that of 1 µm particle by a factor of 5. According to Eq. (7) in Supplementary Information, one then expects that the thermophoretic velocity of 5 µm particle is comparable to that of 1 µm particle^55^. On the other hand, it is known that the dielectrophoretic force is scaled to the cubic power of particle size $(i.e.,\langle {\overrightarrow{F}}_{DEP}\rangle \propto {a}^{3})$ (see Eq. (6) in Supplementary Information). An increase of particle size from 1 µm to 5 µm would amplify the dielectrophoretic force 125 times. The resulting dielectrophoretic velocity of 5 µm particle then becomes 25 times of that of 1 µm particle (as shown by Eq. (5) in Supplementary Information). In other words, the dielectrophoretic velocity of 5 µm particle is enhanced by one order of magnitude as compared to 1 µm particle.

It is noted that from Fig. 6 that the thermophoretic velocity of 1 µm particle is nearly two orders of magnitude higher than its corresponding dielectrophoretic velocity. Considering this fact and the aforementioned discussion, we can conclude that the thermophoretic velocity of 5 µm particle is still at least one order of magnitude higher than its corresponding dielectrophoretic velocity. Consequently, the thermophoresis still plays a more important role than the dielectrophoresis in the enrichment of 5 µm particles. With further increment of the particle size, the dielectrophoresis would overtake the thermophoresis, and there would be eventually a transition from the thermophoresis-dominated enrichment to the dielectrophoresis-dominated enrichment. This is very different from the conventional conception that the enrichment of particles at microfluidic constrictions is only dominated by dielectrophoresis^57, 58^.

The oscillation of trapped 5 µm particles near the constriction is due to a force imbalance on particles induced by the predominant thermophoretic effect. Under the same AC electric field, as increasing the DC electric field from 700 V to 800 V, the enhancement of both the electrophoretic motion of particles (from the left to the right) and the thermophoretic trapping force on the particles can lead to the particle trapping on the right side of constriction as shown in Fig. 9(b). Two movies are submitted as the Supplementary Information for Fig. 9(a) and (b).

## Conclusions

In this work, we have reported both experimental and numerical investigation of the enrichment of colloidal particles in a PDMS microchannel with a convergent-divergent structure. To our best knowledge, this is the first experiment demonstrating the particle enrichment with the Joule heating induced temperature gradient focusing method. In particular, the enrichment of colloidal particles was achieved by our proposed combined AC and DC field technique. Our experiments showed that the use of a combined AC and DC is superior to the use of a sole DC field. Furthermore, it was found that for a fixed AC voltage, there exists a window of applied DC voltages that can achieve successful particle enrichment. However, outside this DC voltage window, a lower DC voltage cannot produce sufficient Joule heating to induce temperature gradient required for enriching particles, and a higher DC voltage leads to fast motion of particles such that particles are swept away, with no enrichment at the constriction of the microchannel. In such case, a higher AC component is needed to produce a higher temperature gradient for TGF of particles.

Four modes of particle transport have been considered in the Joule heating induced TGF of particles, including advective motion of particles due to electroosmotic bulk flow, electrophoretic motion of particles, dielectrophoretic motion of particles and thermophoretic motion of particles. The experimental results suggest the electrokinetically driven TGF of particles is mainly ascribed to the thermophoresis for the particle size considered in this study. Due to the cubic power scaling of the force with particle size, the dielectrophoresis would dominate the particle enrichment as the particle size further increases beyond a critical value. The advection and electrophoresis of particles mainly contribute to transporting particles from the reservoirs to the zone of enrichment.

A numerical model is also formulated to describe the Joule heating induced TGF of particles under a combined AC and DC field. The numerical simulations can reproduce the experimental results reasonably well. Furthermore, the proposed numerical model allows gaining more physical insights into the observed particle enrichment by quantitatively providing the velocity profiles of the abovementioned four particle transport modes, which are apparently not accessible from the experiments. In addition, the numerical simulations verify that the existence of thermophoresis in the Joule heating induced TGF of relatively small particles not only enhances the particle enrichment but also helps to define the shape and location of the zone of particle enrichment.

## Method

### Equipment setup

**Figure 10:**
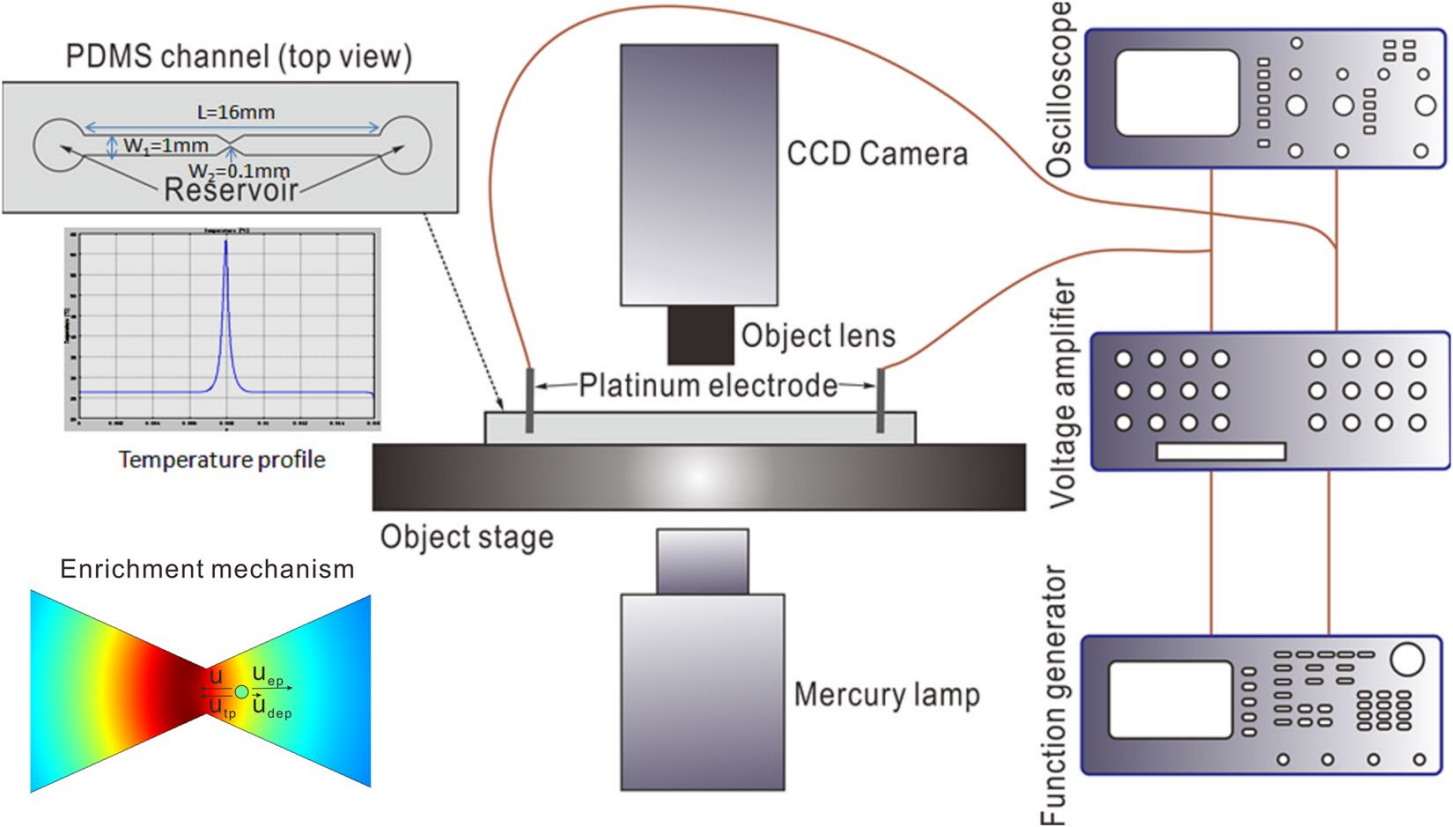
Schematic of the entire experimental setup. A PDMS microfluidic channel with a convergent-divergent structure is loaded with fluorescent particles dispersed in a buffer solution, and is placed on the microscope objective stage. Two platinum electrodes placed in each reservoir of the microchannel are for supplying the required voltages provided by a function generator together with an amplifier. The electrically produced Joule heat in the solution couples with the convergent-divergent structure to give rise to significant axial temperature gradient near the narrowest constriction of channel, as is evident from the inset showing the axial temperature profile. During the experiments, the mercury lamp illuminates the fluorescent particles, and the recording of particle enrichment is through an objective lens and a CCD camera. The bottom-left portion presents a schematic of particle enrichment due to four particle transport mechanisms, including the advection of particle due to electroosmotic flow of buffer solution (u), electrophoresis of particle (u_ep_), dielectrophoresis of particle (u_dep_) and thermophoresis of particle (u_tp_).

The setup of entire experimental system is shown in Fig. 10. A fluorescence microscope (Carl Zeiss, Germany) equipped with a mercury lamp (mbq 52 ac, Zeiss) for excitation of fluorescent particles was used in the experimental investigation. The microfluidic device for concentrating microparticles consists of a straight PDMS channel with a convergent-divergent structure in the middle section of channel (see details in the top-left portion of Fig. 10). The PDMS channel with a length of 16mm, a height of 50 µm, a width of 1mm was fabricated using the standard soft lithography. The length of microfluidic convergent-divergent structure is 2mm, with the narrowest constriction being 100 µm. Two reservoirs with a diameter of 6mm were punched at the two ends of channel for introducing particle samples. Two platinum wires of 0.5 mm in diameter (Sigma-Aldrich, USA) placed in each reservoir were used for applying electrical voltages. To produce combined AC and DC voltages, the AC signal with DC bias was generated by a function generator (AFG3102 Dual Channel Arbitrary/Function Generator, Tektronix Inc.), and then was amplified to the required combined AC and DC voltage by a customized three-channel voltage amplifier (OPT3-AC800-DC1.5 K, Optrobio Technologies Pte Ltd). In order to maintain the accuracy of the input voltage to the microfluidic device, an oscilloscope (100 MHz CombiScope® HM1008-2, HAMEG Instruments) was used to monitor the voltage output from the amplifier. A power source (PS350, Stanford Research System, USA) was used for supplying pure DC voltages. The enriching phenomena were monitored through a 5x objective lens and then recorded with a CCD camera (Sensovation AG, Germany) at a rate of 15 frames per second.

The bottom-left portion of Fig. 10 presents the particle enrichment due to four transport mechanisms. As is shown, Joule heat generated in the conducting buffer couples with the varying channel cross-section to produce an axial temperature gradient. In the presence of electric field, electroosmotic flow of buffer solution is directed from the right to the left, dragging particle inside the solution to move with a velocity of u. The particle electrophoresis moves the particle from the left to the right with a velocity of u_ep_, which is opposite to the electroosmotic bulk flow. The dielectrophoresis repels the particle from the region of high electric field strength towards the region of low electric field strength with a velocity of u_dep_. The thermophoresis induces the motion of particle from the cold region to the hot region with a velocity of u_tp_ due to the strong temperature gradient produced by Joule heating. The imbalance of four particle transport mechanisms (u, u_ep_, u_dep_, u_tp_) results in a net particle motion, and it directs particles to the constriction from both sides, thereby leading to the enrichment of particles at constriction.

### Sample preparation

Green fluorescent particles of 500 nm, 1 µm and 5 µm (Thermo Fisher Scientific Inc.) were used in particle enrichment experiments. Because of the Thermo Fisher Scientific’s Firefli™ process, these florescent particles are highly stable and their fluorescence intensity is insensitive to the temperature change within the range of our experiment. Prior to the experiment, each original particle suspensions (1% w/w solids) were all diluted with a Tris-borate buffer solution (180 mM until specified otherwise) for 500 times volumetrically. Before being loaded into the microfluidic channel, all prepared colloidal samples were sonicated for 15 minutes in an ultrasonic cleaner (Elmasonic E30H, Elma Ultrasonics) for homogenization. Particularly, the Tris-borate buffer prepared from Tris(hydroxymethyl)aminomethane (Sigma-Aldrich, USA) and borate acid (Sigma-Aldrich, USA) was purposely used to maintain pH of the electrolyte medium.

### Experimental procedure and image analysis

The microchannel with two reservoirs was first flushed with deionized water and then filled with the experimental sample solutions. After the electrodes were placed into the reservoirs, the buffer solution levels in two reservoirs were carefully adjusted for balance. This procedure ensures no induced flow by pressure difference resulting from the solution level difference in two reservoirs. Subsequently, the mercury arc lamp was turned on to illuminate the fluorescent particles. The power supply was then immediately switched on to generate electric field along the channel. At the same time, the grayscale fluorescent images were captured by the CCD camera and simultaneously recorded into a personal computer for subsequent analyses. In this study, two different modes of applied voltages, i) a combined AC and DC voltage, and ii) a sole DC voltage were tested as the applied electric field. For a combined AC with DC voltage, the AC component was fixed at 800 V (peak to peak voltage) which is the maximum AC voltage that can be supplied by our current voltage amplifier, and the DC bias was varied from 600 V to 900 V. For the case of sole DC voltages, it was varied from 700 V to 1100 V. For the sake of quantifying the enhancement of particle concentration, the grayscale value of microscopic images was extracted with a MATLAB program and subsequently converted to the concentration of particles by using the calibration curves provided in ref. 48.

## Electronic supplementary material

Supplementary Information

Movie for Figure 3

Movie for Figure 4a

Movie for Figure 4b

Movie for Figure 9a

Movie for Figure 9b
