# Supplementary material for: Electrokinetically driven continuous-flow enrichment of colloidal particles by Joule heating induced temperature gradient focusing in a convergent-divergent microfluidic structure
Source: Sci Rep. 2017 Sep 7;7:10803. doi: 10.1038/s41598-017-11473-w (PMC5589950; doi:10.1038/s41598-017-11473-w)
Supplement: Supplementary file 1 — Supplementary Information [file 41598_2017_11473_MOESM1_ESM.docx]

**Supplementary Information**

**Electrokinetically driven continuous-flow enrichment of colloidal particles by Joule heating induced temperature gradient focusing in a convergent-divergent microfluidic structure**

Cunlu Zhao ^1,*^, Zhengwei Ge ^2,*^, Yongxin Song ^3^ & Chun Yang ^2^

^1^ Key Laboratory of Thermo-Fluid Science and Engineering of MOE, School of Energy and Power Engineering, Xi’an Jiaotong University, Xi’an 710049, China

^2^ School of Mechanical and Aerospace Engineering, Nanyang Technological University, 50 Nanyang Avenue, Singapore 639798

*^3^ Department of Marine Engineering, Dalian Maritime University, 1 Linghai Road, Dalian 116026, China*

*These authors contributed equally to this work.

Correspondence should be addressed to C.Z. ([mclzhao@mail.xjtu.edu.cn](mailto:mclzhao@mail.xjtu.edu.cn)) or C.Y. ([mcyang@ntu.edu.sg](mailto:mcyang@ntu.edu.sg)).

**Numerical model**

In the Joule-heating mode of TGF, the application of an external electric field directly gives rise to three electrokinetic mechanisms of particle transport, and they are advective transport of particle due to the bulk electroosmotic flow of liquids, electrophoresis of particles, and dielectrophoresis of particles. Furthermore, the passing of electrical current also generates a temperature gradient due to the change of electric current density induced by the varying cross-section of microchannel. Such induced temperature gradient on one hand modifies the abovementioned three electrokinetic mechanisms of particle transport (bulk electroosmosis, electrophoresis of particles and dielectrophoresis of particles), and on the other hand introduces another mechanism of particle transport, i.e., thermophoresis. It is the combination of four mechanisms that govern the transport of particles. Therefore, the model should consist of governing equations for the mass transport of particles (concentration), the electric potential, the temperature field and the bulk electroosmotic flow field. Among these governing equations, the electric field governed by the Laplace equation is pseudo-steady. It was also previously shown that the time scales of the governing equations for liquid flow and temperature are usually about three orders of magnitude smaller than that of the mass transport equation [^1^](#_ENREF_1), so the temperature and velocity fields also can be assumed to be pseudo-steady and consequently only the mass transport of particles dominates the transient behavior of particle enrichment.

**a. Transient mass transport equation describing the electrokinetic enrichment of particles.** In our previous work, the model development considered the concentration of solute by Joule-heating mode of TGF [^1^](#_ENREF_1). Specifically, the solute transport is due to electroosmosis-induced advection and electrophoresis, without the contribution of dielectrophoresis and thermophoresis. This is because the velocity of small solute molecules (of size about $O(10^{-9})\text{ m}$) due to these two mechanisms is of about $O(10^{-6})\text{ m/s}$ which is three orders of magnitude smaller than that due to electrophoresis and the electroosmosis-induced advection (~$O(10^{-3})\text{ m/s}$). The present investigation involves the concentration of particles (around 1µm) much larger than solute molecules, and thus dielectrophoresis and thermophoresis play important roles. Here we take into account the contributions of dielectrophoresis and thermophoresis to the particle transport. With the assumptions of no adsorption of particles onto the microchannel wall and no interaction among particles, the particle concentration is described by the mass transport equation as

$\frac{\partial C}{\partial t}+\nabla\cdot\left(\vec{u}C+\vec{u}_{ep}C+\vec{u}_{dep}C+\vec{u}_{tp}C\right)=\nabla\cdot\left[D\left(T\right)\nabla C\right]$ (1)

where $C$ is the concentration of particles, and $D\left(T\right)$ is the temperature-dependent mass diffusivity of particles and can be estimated from the [Stokes*-*Einstein](http://arxiv.org/abs/1211.0686)relation

$D\left(T\right)=\frac{k_{B}T}{6\pi\eta\left(T\right)a}$ (2)

where *k*_B_ is the Boltzmann constant, *a* is the particle radius, and $\eta\left(T\right)$ is the temperature-dependent viscosity of buffer solution and is characterized by the relationship, $\eta\left(T\right)=3.96\times10^{-8}T e^{\frac{708.17}{T-134.48}}$. Eq.(1) clearly indicates five mechanisms of the transport of particles, namely, the bulk flow advection $\vec{u}C$, electrophoretic transport $\vec{u}_{ep}C$, dielectrophoretic transport $\vec{u}_{dep}C$, thermophoretic transport $\vec{u}_{tp}C$, and diffusive transport $D\left(T\right)\nabla C$.

The electrophoretic velocity of particle, $\vec{u}_{ep}$ can be simply expressed as

$\vec{u}_{ep}=\mu_{ep}(T)\vec{E}(T)$ (3)

where $\mu_{ep}\left(T\right)$ and $\vec{E}(T)$ are the temperature-dependent electrophoretic mobility of particles and electric field, respectively. The electrophoretic mobility can be expressed as

$\mu_{ep}\left(T\right)=\varepsilon_{0}\varepsilon_{m}\left(T\right)\zeta_{p}\left(T\right)/\eta\left(T\right)$ (4)

where ε_0_$\varepsilon_{0}$ is the electric permittivity of vacuum, and ε_m_(T)$\varepsilon_{m}\left( T \right)$ is the temperature-dependent dielectric constant of buffer solution and can be expressed as, $\varepsilon_{m}\left( T \right)=308.56e^{-4.6\times{10}^{-3}T}$. It is known that charge (or zeta potential) of solid surfaces is governed by the dissociation of chemical groups. The more significant the dissociation is, the higher charge density or zeta potential the surface acquires. Qualitatively, the increase of temperature causes the surface groups to dissociate more, and then leads to the increment of the surface charge or zeta potential. Furthermore, as temperature can also affect other factors such as pH, double layer thickness and solution dielectric constant etc, the dependence of zeta potential on temperature is a quite complicated matter. The temperature-dependent zeta potentials of particles and channel walls can be evaluated from an empirical relation [^2^](#_ENREF_2), $\zeta_{i}\left( T \right)=\zeta_{i}\left( T_{0} \right)\left[ 1+0.004\left( T-T_{0} \right) \right]$ with T_0_ being room temperature and the subscript *i*=w (p) denoting channel walls (particles).

The dielectrophoretic velocity of particles, $\vec{u}_{dep}$ can be expressed as

$\vec{u}_{dep}=\frac{\left\langle\vec{F}_{DEP}\right\rangle}{6\pi\eta\left(T\right)a}$ (5)

where $\left\langle\vec{F}_{DEP}\right\rangle$ represents the time-averaged dielectrophoretic force. In this work, as the conductivity of polystyrene latex particles is much lower than that of the surrounding Tris-borate buffer solution, particles experience a negative dielectrophoretic force which transports the particles from the region of high electric field strength to the region of low electric field strength. The dielectrophoretic force exerting on a spherical particle can be expressed as

$\left\langle\vec{F}_{DEP}\right\rangle=-\pi\varepsilon_{0}\varepsilon_{m}\left(T\right)a^{3}\nabla\left(\vec{E}^{2}_{dc}+\vec{E}^{2}_{rms,ac}\right)$ (6)

where *E*_dc_ is the DC electric field strength and $E_{AC,rms}$ is the root mean square of AC electric field strength. In the context of microfluidics, dielectrophoresis is mainly for manipulation, separation and concentration of particles and cells, and usually complex metal electrodes and channel structures are needed.

The thermophoretic velocity of particles, $\vec{u}_{tp}$ can be expressed as

$\vec{u}_{tp}=-S_{T}D\left(T\right)\nabla T$ (7)

where S_T_ is the Soret coefficient. Usually, when the particles migrate from the low temperature to the high temperature regions (so-called thermophilic behavior), S_T_ takes negative values; while when the particles migrate from the high temperature to the low temperature regions (so-called thermophobic behavior), S_T_ takes positive values.

For the boundary conditions associated with the mass transport, no mass flux boundary condition and initial particle concentration are specified on the channel walls and at the inlet/outlet boundaries, respectively.

**b. Externally applied electric field.** In order to evaluate the three electrokinetic mechanisms of particle transport and the temperature gradient, the electrical field inside the microchannel is required. For the electrolyte buffer used in the present experiment, the electric double layer (EDL) is estimated to be of about several nanometers which is much smaller than the dimension of the microchannel (of about 100µm). The presence of such thin EDL can lead to the use of a slip velocity approach with the electrolyte solution in the bulk channel considered as electrically neutral. Then the electric field in microchannel, , is governed by the law of electric current conservation which can be mathematically described by the Laplace equation as

$\nabla\cdot\left[\lambda\left(T\right)\nabla\varphi\right]=0$ (8)

where $\lambda\left(T\right)$ is the temperature-dependent electrical conductivity of the electrolyte buffer solution and can be expressed as ${\lambda\left( T \right)=\lambda}_{0}\left[ 1+0.02\left( T-T_{0} \right) \right]$ for electrolyte solutions ^41^; here $\lambda_{0}$ is the conductivity of electrolyte at room temperature $T_{0}$, and can be measured in the experiments for different buffer concentrations.

The boundary conditions for Eq. (8) include the specified voltages on the channel inlet and outlet and the electrically insulating boundary condition on the channel walls.

**c. Pseudo-steady temperature field induced by Joule heating.** The external electric field induces temperature gradient due to Joule heating. The corresponding temperature field in the microfluidic channel is governed by the energy equation expressed as

$\rho c_{p}\vec{u}\cdot\nabla T=k_{l}\nabla^{2}T+\lambda\left(T\right)\vec{E}(T)\cdot\vec{E}(T)$ (9)

where *ρ*, $c_{p}$ and $k_{l}$ are the density, specific heat and thermal conductivity of the buffer solution, respectively. These three properties of buffer solutions are marginally dependent on temperature and thus are assumed to be constant in the simulation. The temperature-dependent electric field strength can be evaluated as, .

Since the thermal conductivity of channel wall (PDMS) is significantly lower than that of buffer solution, the thermal insulation boundary condition is assumed on the channel walls. The inlet boundary is given as the room temperature and the outlet boundary is assumed to reach the fully-developed thermal convection state.

**d. Pseudo-steady bulk flow field due to electroosmosis.** The flow information of bulk flow due to electroosmosis much be known when calculating the advective particle transport in Eq. (1). As mentioned above, the EDL thickness of the channel wall is significantly thinner than the channel dimensions, and thus the variation of fluid velocity within the thin EDL can be neglected. This thin EDL situation allows us to model the bulk flow with a slip velocity approach. For an incompressible buffer solution, the bulk fluid velocity field due to electroosmosis is described by the Navier-Stokes equation and the continuity equation

$\rho\vec{u}\cdot\nabla\vec{u}=-\nabla p+\nabla\cdot\left\{\eta\left(T\right)\left[\nabla\vec{u}+\left(\nabla\vec{u}\right)^{T}\right]\right\}$ (10)

$\nabla\cdot\vec{u}=0$ (11)

where *p* is the pressure, and the channel walls are assumed to slip with the Helmholtz-Smoluchowski velocity

$\vec{U}_{s}=-\frac{\varepsilon_{0}\varepsilon_{m}\left(T\right)\zeta_{w}\left(T\right)\vec{E}(T)}{\eta(T)}$ (12)

In experiments, since there is no externally applied pressure difference along the channel, the pressure at the inlet and outlet boundaries is set to be zero.

**e. Modifications of governing equations under a combined AC and DC electric field.** The above governing equations are generally applicable when a sole DC electric field is applied. In this study, a combined AC and DC electric field is used for Joule-heating mode of TGF, and hence following modifications should be made to respective equations:

(i) *Modification of the mass transport equation*. Since the frequency of applied AC in experiments was maintained 10kHz, the corresponding time period of AC field is $\tau_{AC}\sim O\left( {10}^{-4} \right)$s. The characteristic time of the mass transport, $\tau_{C}=D_{h}^{2}/D\left( T_{0} \right)$ is estimated to be about $\tau_{C}\sim O\left( {10}^{2} \right)$s (here D_h_ is the hydraulic diameter of the microchannel). Clearly based on the fact that $\tau_{AC}{\ll\tau}_{C}$, the AC field component does not contribute to the electrophoretic velocity of particles $\vec{u}_{ep}$ used in the mass transport equation, and hence $\vec{u}_{ep}$ is only driven by the DC component of electric field, namely $\vec{u}_{ep}=\mu_{ep}\left(T\right)\vec{E}_{DC}$.

(ii) *Modification of the governing equation of electric field*. The introduction of an AC electric field makes the externally applied field become time-dependent. However, as is shown earlier, the time period of the AC signal, $\tau_{AC}$, is much smaller than the characteristic time of the mass transport equation, $\tau_{C}$. As a result, the electric filed can be solved by using a pseudo-steady approach, and the obtained electric potential is a root-mean squared value of the combined AC and DC electric field.

(iii) *Modification of the governing equation of temperature*. The Joule-heating term in the governing equation of temperature needs to be modified. The combined AC and DC electric field can be expressed as $\vec{E}=\vec{E}_{AC}+\vec{E}_{DC}=\vec{A}\sin\left(2\pi ft\right)+\vec{B}$ (where *f* is the frequency of AC signal, $\left|\vec{A}\right|$ and $\left|\vec{B}\right|$ are the magnitude of AC and DC field strength, respectively). Then the time-averaged Joule-heating term within one time period can be derived as

$\overline{\lambda\vec{E}\cdot\vec{E}}=\frac{\lambda}{1/f}\int_{0}^{1/f} \vec{E}\cdot\vec{E}dt=\lambda\left|\frac{\vec{A}}{\sqrt{2}}\right|^{2}+\lambda\left|\vec{B}\right|^{2}=\lambda E_{AC,rms}^{2}+\lambda E_{DC}^{2}$ (13)

which indicates that the Joule heat includes contributions of both AC and DC components of the applied electric field. Hence, to generate the same amount of Joule heat as the case of a sole DC field, the required DC voltage can be much reduced when an AC component is introduced. On the other hand, even if the AC component can produce sufficient amount of Joule heat to generate a required temperature gradient for TGF, the DC component is still needed for transport of the particles.

(iv) *Modification of the governing equation of flow field*. The slip velocity determined by the Helmholtz-Smoluchowski equation should be modified for the governing equation of the bulk flow field. It is noted that the characteristic response time of bulk electroosmosis after switching on the electric field can be estimated from $\tau_{eof}=O\left[D_{h}^{2}\rho/\eta\left(T_{0}\right)\right]$. The estimation shows that the response time is of order about $\tau_{eof}\sim O\left(10^{-2}\right)$s. In experiments, an AC frequency of 10 kHz was chosen such that the time period of AC electric field is about $\tau_{AC}\sim O\left( {10}^{-4} \right)$ s which is two orders of magnitude smaller than $\tau_{eof}$. Hence, similar to the case of the particle electrophoretic velocity $\vec{u}_{ep}$, only the DC component contributes to the bulk flow, and the Helmholtz-Smoluchowski slip velocity on the channel walls can then be rewritten as

$\vec{U}_{s}=-\frac{\varepsilon_{0}\varepsilon_{m}\left(T\right)\zeta_{w}\left(T\right)\vec{E}_{DC}}{\eta\left(T\right)}$ (14)

The set of modified governing equations discussed above was solved numerically using the commercial software Comsol Multiphysics 4.3, and the details of execution of numerical modelling can be found in our previous work.[^1^](#_ENREF_1)

**References**

1 Ge, Z., Yang, C. & Tang, G. Concentration enhancement of sample solutes in a sudden expansion microchannel with Joule heating. *Int. J. Heat Mass Transfer* **53**, 2722-2731, doi:10.1016/j.ijheatmasstransfer.2010.02.033 (2010).

2 Sommer, G. J., Kim, S. M., Littrell, R. J. & Hasselbrink, E. F. Theoretical and numerical analysis of temperature gradient focusing via Joule heating. *Lab Chip* **7**, 898-907, doi:10.1039/B701894K (2007).
